# Commentary: Cooperation Not Competition: Bihemispheric tDCS and fMRI Show Role for Ipsilateral Hemisphere in Motor Learning

**DOI:** 10.3389/fnhum.2018.00097

**Published:** 2018-03-15

**Authors:** Brenton Hordacre, Mitchell R. Goldsworthy

**Affiliations:** ^1^Sansom Institute for Health Research, School of Health Sciences, University of South Australia, Adelaide, SA, Australia; ^2^Robinson Research Institute, School of Medicine, University of Adelaide, Adelaide, SA, Australia; ^3^Discipline of Psychiatry, School of Medicine, University of Adelaide, Adelaide, SA, Australia

**Keywords:** motor learning, motor cortex, plasticity, transcranial direct current stimulation, functional magnetic resonance imaging

Therapy to facilitate motor learning requires *a priori* knowledge of the motor system. The “interhemispheric competition” model posits that the contralateral hemisphere suppresses activity of the ipsilateral hemisphere to reduce putative interference of ipsilateral descending pathways thought to degrade motor performance. Non-invasive brain stimulation paradigms are well positioned to test models of motor control. For instance, transcranial direct current stimulation (tDCS) applied to the human motor cortex (M1), can induce polarity-dependent changes in corticospinal excitability that outlast the period of stimulation. Therefore, facilitatory anodal tDCS of the contralateral hemisphere and/or inhibitory cathodal tDCS of the ipsilateral hemisphere should enhance motor learning. While the interhemispheric competition model has guided therapeutic application of tDCS in neurorehabilitation (Di Pino et al., 2014), there is some evidence supporting a role of the ipsilateral hemisphere in shaping motor output (Verstynen et al., 2005; Cabibel et al., 2018). As a result, the interhemispheric competition model may be oversimplified or partially inaccurate and requires further investigation.

Recently, Waters et al. (2017) investigated the role of the ipsilateral hemisphere in learning of a sequential key-press task. Subjects were pseudo randomized to one of four tDCS groups: unihemispheric (anode contralateral M1, cathode ipsilateral supraorbital ridge), conventional bihemispheric (anode contralateral M1, cathode ipsilateral M1), reverse-polarity bihemispheric (anode ipsilateral M1, cathode contralateral M1), and sham. Stimulation (2 mA) was applied over 4 consecutive days for the first 25 min of a ~60 min training session. Conventional and reverse-polarity bihemispheric stimulation resulted in learning improvements beyond that observed following unihemispheric and sham stimulation. Functional magnetic resonance imaging (fMRI) found both conventional and reverse-polarity bihemispheric tDCS increased task-related activation of contralateral and ipsilateral hemispheres relative to sham. It was therefore suggested that bihemispheric tDCS, irrespective of polarity, led to similar improvements in motor learning and increased neural activation in both hemispheres, supporting “interhemispheric cooperation” as opposed to “interhemispheric competition.”

While the conclusions of Waters et al. (2017) were supported by both behavioral and functional neuroimaging data, the direct interpretation of the latter, at least, should be further examined. FMRI blood oxygenation level-dependent (BOLD) responses provide an indirect measure of neural activity, and therefore cannot readily distinguish between excitatory and inhibitory synaptic activity (Arthurs and Boniface, 2002). In support, a previous study found anodal and cathodal tDCS both increased task-related BOLD activity despite having facilitatory and inhibitory effects on corticospinal excitability, respectively (Stagg et al., 2009). It may be that both facilitatory and inhibitory synaptic activity increase BOLD response through a cascade of events at cellular and molecular levels with long-lasting after-effects mediated by a shift in metabolically demanding NMDA and GABA receptor activity (Arthurs and Boniface, 2002). These tDCS induced effects may last for a number of days and could explain why Waters et al. (2017) observed increased BOLD activity for both conventional and reverse-polarity bihemispheric stimulation.

Nevertheless, both conventional and reverse-polarity bihemispheric tDCS induce similar improvements in motor learning. To explain this observation, Waters et al. (2017) propose that the effects of stimulation may have been polarity-unspecific since the response to stimulation was similar despite reversal of polarity. While acknowledging that the inclusion of transcranial magnetic stimulation (TMS) to quantify changes in cortical excitability by recording motor evoked potentials (MEPs) following tDCS would help confirm this suggestion, there may be additional explanations which require consideration. Although convention suggests anodal stimulation increases and cathodal stimulation decreases excitability, responses are known to be variable in magnitude and direction. Higher intensity and/or longer duration of stimulation can modulate or reverse tDCS response (Monte-Silva et al., 2013; Jamil et al., 2017). Interestingly, previous studies have reported bihemispheric tDCS applied for 15 min at 1–1.5 mA resulted in the expected polarity specific modulation of excitability in each hemisphere (Goodwill et al., 2013; Tazoe et al., 2014). It may be that the higher intensity (2 mA) or longer duration of stimulation (25 min) used by Waters et al. (2017) has caused tDCS after-effects to differ from the canonical modulation of excitability. Furthermore, the susceptibility of tDCS after-effects to inter- and intra-individual sources of variability bears consideration. Briefly, plasticity responses following brain stimulation can be modulated by numerous factors, including anatomical and functional properties of the stimulated network, age, genetics, pharmacology, and circadian rhythms (Ridding and Ziemann, 2010; Hordacre et al., 2017a,b). Given the potential for variability in response to tDCS, quantifying changes in corticospinal excitability would provide evidence to confirm that the anticipated response to stimulation has occurred.

To further examine the proposed interhemispheric cooperation model, we suggest additional TMS experimental approaches. First, paired-pulse TMS paradigms can be employed to investigate inhibitory synaptic activity (Figure 1). Both GABA_A_ and GABA_B_ receptor mediated inhibition can be tested using paradigms known as short-latency intracortical inhibition and long-latency intracortical inhibition. Since GABA plays an important role in synaptic plasticity and motor learning (Stagg et al., 2011), probing the role of GABAergic inhibition may uncover further mechanistic information to clarify the response to different current directions and polarities of stimulation. Furthermore, dual-coil paired-pulse TMS can be used to probe excitability of interhemispheric inhibitory pathways (Figure 1). Since the interhemispheric competition and cooperation models differ in the proposed role of the ipsilateral hemisphere, probing the nature of interhemispheric interactions through these pathways appears appropriate.

**Figure 1:**
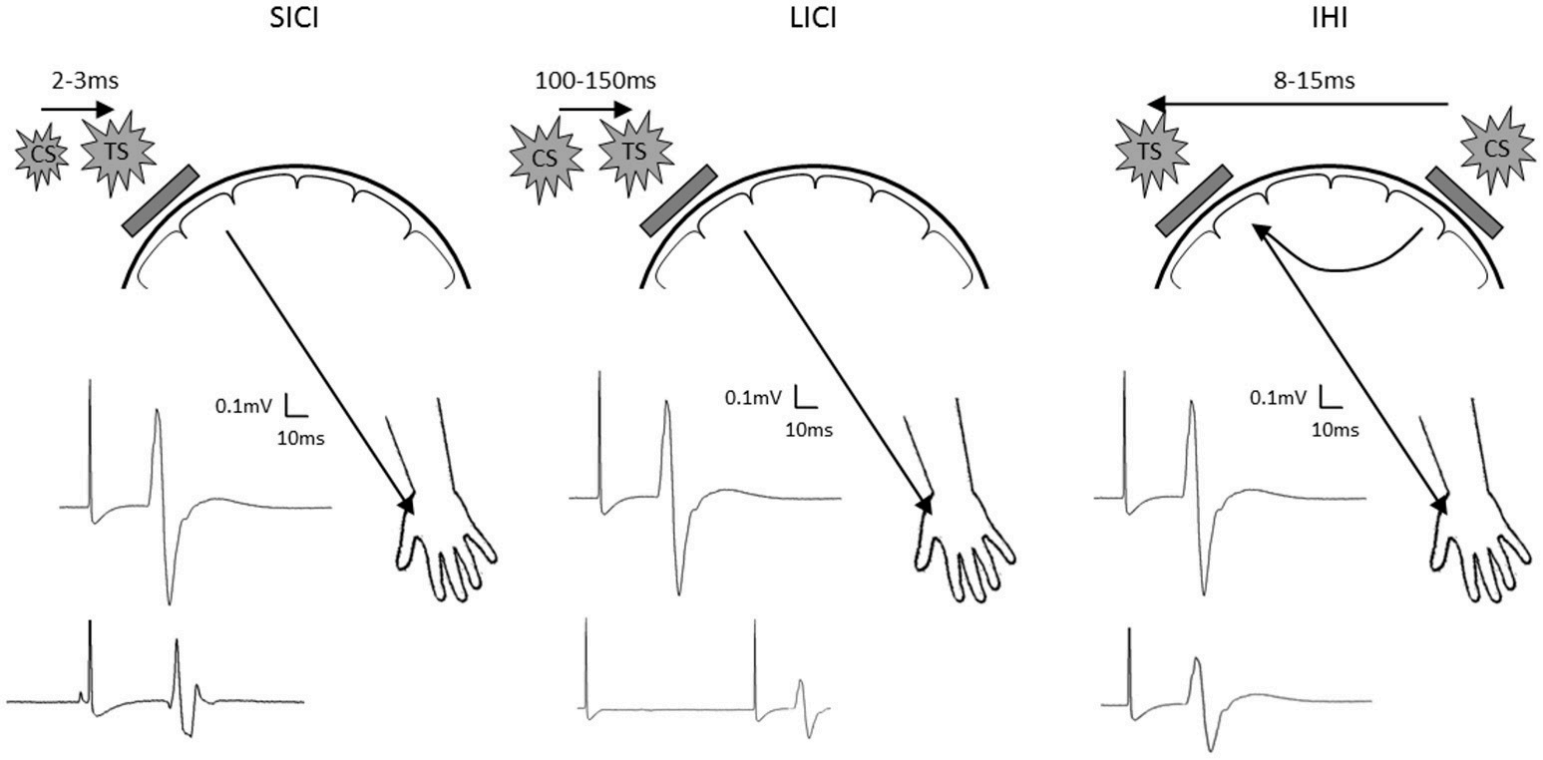
Schematic diagram showing short latency intracortical inhibition (SICI; **left**), long latency intracortical inhibition (LICI; **center**) and interhemispheric inhibition (IHI; **right**). Location of the test stimuli and conditioning stimuli are shown on the schematic diagram of the head. Examples of MEP recorded at the contralateral hand are shown with test stimulus only shown as the top MEP, and the conditioned MEP shown as the bottom MEP. TS, test stimulus; CS, conditioning stimulus.

The recent study from Waters et al. (2017) provides an important set of results which challenge our understanding of human motor control by suggesting that both hemispheres cooperate to facilitate motor learning. Additional experimental work using TMS may provide a more complete picture of the underlying neurophysiology. Additionally, and importantly, the magnitude and direction of tDCS-induced effects in the brain are highly variable, and the factors responsible for this variability are still not completely understood. Nevertheless, while caution is warranted before adopting the framework set out by Waters et al. (2017), their results suggest that traditional views of interhemispheric competition during motor learning needs to be re-evaluated. To this end, techniques such as tDCS and TMS have great potential for uncovering the role of the ipsilateral cortex in motor learning. This may provide new opportunities to assist clinical practice for stroke recovery. For example, preliminary evidence suggests the contralesional hemisphere may be an appropriate therapeutic target in severely impaired stroke survivors (McCambridge et al., 2018). Although requiring further investigation, it may be that therapeutic approaches utilizing the potentially beneficial role of the contralesional hemisphere are able to improve function of the paretic upper limb.

## Author contributions

BH and MG all made substantial intellectual contributions to the manuscript and approved the final version for publication.

### Conflict of interest statement

The authors declare that the research was conducted in the absence of any commercial or financial relationships that could be construed as a potential conflict of interest.
